# Cyanine-Doped Nanofiber Mats for Laser Tissue Bonding

**DOI:** 10.3390/nano12091613

**Published:** 2022-05-09

**Authors:** Fulvio Ratto, Giada Magni, Annalisa Aluigi, Marta Giannelli, Sonia Centi, Paolo Matteini, Werner Oberhauser, Roberto Pini, Francesca Rossi

**Affiliations:** 1 Istituto di Fisica Applicata “Nello Carrara”, Consiglio Nazionale delle Ricerche, Via Madonna del Piano 10, 50019 Sesto Fiorentino, FI, Italy; g.magni@ifac.cnr.it (G.M.); s.centi@ifac.cnr.it (S.C.); p.matteini@ifac.cnr.it (P.M.); r.pini@ifac.cnr.it (R.P.); 2 Istituto per la Sintesi Organica e la Fotoreattività, Consiglio Nazionale delle Ricerche, Via P. Gobetti 101, 40129 Bologna, BO, Italy; annalisa.aluigi@isof.cnr.it (A.A.); marta.giannelli@isof.cnr.it (M.G.); 3 Istituto di Chimica dei Composti Organometallici, Consiglio Nazionale delle Ricerche, Via Madonna del Piano 10, 50019 Sesto Fiorentino, FI, Italy; woberhauser@iccom.cnr.it

**Keywords:** electrospun nanofibers, wound dressing, chitosan, indocyanine green, dermis

## Abstract

In spite of an extensive body of academic initiatives and innovative products, the toolkit of wound dressing has always revolved around a few common concepts such as adhesive patches and stitches and their variants. Our work aims at an alternative solution for an immediate restitutio ad integrum of the mechanical functionality in cutaneous repairs. We describe the fabrication and the application of electrospun mats of bioactive nanofibers all made of biocompatible components such as a natural polysaccharide and a cyanine dye for use as laser-activatable plasters, resembling the ultrastructure of human dermis. In particular, we investigate their morphological features and mechanical moduli under conditions of physiological relevance, and we test their use to bind a frequent benchmark of connective tissue as rabbit tendon and a significant case of clinical relevance as human dermis. Altogether, our results point to the feasibility of a new material for wound dressing combining translational potential, strength close to human dermis, extensibility exceeding 15% and state-of-art adhesive properties.

## 1. Introduction

The treatment of accidental or surgical wounds has been resting for centuries on methods and supplies as familiar as sutures and band-aids. What plays in favor of these tools is an indisputable history of success in contexts as diverse as surgical rooms and household medication. However, their deployment also entails problems that combine to complicate the healing process, such as foreign body reactions, microbial infections and leakage of fluids, or that hinder their use in anatomical sites of difficult access such as various cases in ophthalmology, microvascular surgery and neurosurgery. For these reasons, the quest for alternative concepts of wound dressings has always remained a lively field of fundamental and translational research [1,2,3,4,5,6,7].

Our work aims at the possibility of impermeably sealing surgical wounds and immediately restoring the mechanical functionality of a connective tissue in the broadest variety of clinical scenarios. In particular, here, we focus on a new generation of biocompatible patches, where, rather than a chemical glue, the adhesive process originates from laser bonding to a biological target. The general scheme that we have already proposed elsewhere [3] consists of a porous scaffold made of a hybrid mat or sponge containing a bioactive component—a polysaccharide such as hyaluronan [8] or chitosan [9,10,11,12,13,14]—and hosting one of several dyes, such as Food and Drug Administration (FDA)-approved indocyanine green (ICG) [11,14] or inert plasmonic nanoparticles [8,9,10,12,13]. This overall format is suitable to support complementary applications in different regimes of optical activation or interrogation [13]. The case of laser bonding typically requires ms-long pulses or cw light to trigger a cascade of photothermal and biochemical events that produce an adhesive effect at the interface with the connective tissue, whether it be corneas, tendons, vessels or dura mater [10,11,14], for instance. The underlying mechanism is that the embedded dyes mediate a photothermal conversion, and that the base polysaccharide undergoes a process of breaking and reforming inter-molecular bonds that eventually involve the endogenous proteins [9].

Among the various dyes, we have recently shown that innovative materials such as plasmonic nanoparticles of complex architecture, such as Au @ Ag @ polyethylene glycol core @ shell @ shell nanorods (about 100 nm in length × 20 nm in diameter), may simultaneously serve as sensors and actuators covering the functions of photothermal transducer, colorimetric probe of physiochemical parameters, and complementary source of antimicrobial agents [13]. However, plasmonic materials raise new issues in terms of biocompatibility [15] that still keep their use too far from a translational perspective. Here, we take the opposite direction and target the fastest path for a clinical uptake of laser bonding. With respect to our previous work on hydrogels made of chitosan and ICG [11,14], we address their reformulation into conformable scaffolds that may ensure the pliability and extensibility needed for operation and integration in relevant cases of connective tissue, such as human dermis.

Dermis is the connective stratum interposed between the epidermal and subcutaneous layers of the skin. It is composed of collagen, elastic components and other cellular constituents such as glands, nerve endings and blood vessels [16], and it offers an outstanding example of the tight connection that exists between structure and function in biological tissue [17]. Its stroma hosts both elastic fibers made of elastin with a spring constant around 4 MPa and stiff fibers featuring bundles of hundreds of fibrils made of collagen with a spring constant in the order of 4 GPa, which are able to reorient, slide, disassemble and reassemble in response to the applied strain [18,19,20]. The collagenous fibers exhibit a thickness of few microns, whereas the diameter of the individual fibrils is around 50 nm. The final tissue displays the typical behavior of elastomeric materials and a range of moduli spanning as much as three orders of magnitude from about 0.1 to 140 MPa, which depends on a broad variety of factors, such as anatomical site, direction of the applied strain with respect to the so-called Langer’s lines, age, strain rate and, more in general, method of measurement, with tensile tests returning typical results between about 20 and 40 MPa [21,22]. Representative values for the ultimate strength and the maximum strain of the skin before failure are about 10 to 30 MPa and 40 to 60% [18,19]. In the end, all these features are such as to collectively ensure the ideal extent of flexibility and extensibility needed to accommodate motion.

Here, we take inspiration from the ultrastructure of the dermis [23] and reformulate our dressings as mats of bioactive nanofibers containing chitosan and ICG and made by electrospinning [24,25,26,27,28,29,30,31,32,33].

Over recent years, electrospinning has received substantial attention as a process for additive manufacturing of wound dressings, yielding unique versatility both in terms of microporosity and biochemical composition for drug delivery and cell repopulation [34,35]. In our previous work [13], we introduced blends of chitosan and polyvinyl alcohol (PVA) for electrospinning into thin mats of hybrid nanofibers of an average diameter smaller than 100 nm. We chose mixtures of these ingredients for their history of applications in wound dressing and antimicrobial protection [24,25,26,28,29,30,31,32,36,37,38]. We discussed their manufacturability into functional shapes, and their enhancement with active ingredients of interest in wound healing and monitoring. Meanwhile, the use of PVA raises new issues in terms of stability in operative conditions, such as wet and physiological environments, because of its solubility in aqueous buffers, which incidentally makes it a common choice for use as a sacrificial substrate in additive manufacturing [39,40]. Here, we address the feasibility of cross-linked electrospun blends of chitosan and PVA hosting ICG for laser bonding to human dermis, in terms of translational parameters such as conformability and compatibility with the mechanical features of the biological tissue.

## 2. Materials and Methods

### 2.1. Synthesis of the Films

Chitosan (low MW), PVA (typical MW 84–124 kDa), acetic acid and glutaraldehyde (GLA) were purchased from Sigma Aldrich (Milan, Italy). ICG was obtained from PULSION Medical System (Munich, Germany) and was used as received. In brief, we started from a hybrid paste made by mixing 1 part of an acidic solution containing 2.5% chitosan, 0.05% ICG and 1% acetic acid, and 1.5 parts of an aqueous solution containing 9% PVA. Then, we implemented a process for electrospinning with a home-made setup that is described elsewhere [41]. Optimal parameters for electrospinning proved to be a distance of needle to collector of 12 cm and voltage of 25 kV and a flow rate of the hybrid paste of 30 μL/min. Upon rapid evaporation of water and acetic acid, the final composition of the films is therefore about 16% chitosan and 84% PVA with 0.31% ICG.

For cross-linking, films were cut in squares of an approximate side of 1 cm and then exposed to a saturated vapor of an aqueous solution of 25% GLA at room temperature for 2 h by hanging with plasticine and 10-0 nylon suture thread from Johnson & Johnson (New Brunswick, NJ, USA) inside a sealed vial kept under a fume hood. Before subsequent manipulation, samples were transferred to an open vial and left to degas at room temperature overnight under the same hood.

### 2.2. Morphological Analysis of the Films

Samples were observed under a 435 VP Scanning Electron Microscope (SEM, LEO Electron Microscopy, Cambridge, UK) with an acceleration voltage of 15 kV, current of 100 pA and working distance around 20 mm. Some specimens were inspected after immersion in a physiological buffer and then frozen in liquid nitrogen and freeze-dried in a system from Labconco (mod FreeZone 2.5L-50C, Kansas City, MO, USA) before subsequent processing. Before SEM analysis, a part of each sample was sputter-coated with a Au layer using a K550 device from Emitech (Ashford, UK) with a current of 20 mA for an interval of 240 s. Fiber diameters were analyzed by means of GIMP 2.8 (GNU Image Manipulation Program) by taking 100 measurements from random locations and different micrographs.

### 2.3. Mechanical Analysis of the Films

For tensile testing, films were cut in pieces of an approximate size of 6 mm length × 3 mm width and secured with appropriate grips in a custom-made tensiometer (Asper, Florence, Italy) [10] operating at a strain rate of 1 min${}^{-1}$. Some samples were tested under wet conditions upon hydration in physiological buffer for 30 min within a tank purposely designed for compatibility during the measuring run. The mechanical moduli (E) and the ultimate tensile strength (UTS) of the films were calculated by dividing the applied load by their steric section, which was measured as the product of width times average thickness. The latter was quantified by sampling multiple locations of the as-spun mats before cross-linking and by implementing a confocal laser scanning microscope equipped with a motorized stage (mod TCS SP8, Leica Microsystems, Mannheim, Germany) operated in reflectance mode at 63× magnification. The result was (11 ± 2) μm, and it was taken as a fixed value throughout this work.

All measurements were repeated at least 5 times per kind of sample, in order to assess their reproducibility.

### 2.4. Protocols for Laser Bonding to Rabbit Tendons and Human Dermis

The feasibility of the different scaffolds for laser bonding was tested in rabbit tendons and human dermis. The former is a common benchmark in laser bonding [10,13], whereas the latter is the principal target of this work. Freshly harvested tendons from rabbit legs were purchased from the local slaughterhouse and used within 4 h from sacrifice. Human dermis unsuitable for transplantation was obtained from Banca dei Tessuti e Terapia Tissutale (ASST, Grande Ospedale Metropolitano Niguarda, Milan, Italy). A diode laser emitting cw light at 810 nm (mod SMARTY A800, DEKA, Calenzano, Italy) was used to weld the films onto the tendons or the skin, according to our previous publications [1,14,42,43,44]. In brief, laser light was coupled into a multi-mode 300 μm-core diameter optical fiber terminating in a handpiece. The treatment was performed under a surgical microscope (Takagi Ophthalmic Instruments Europe, Manchester, UK). The fiber optic tip was kept in contact with the films that were gently pressed onto the tendons or the dermis in order to ensure their intimate contact and to gain as much control as possible over the size of each spot against the beam divergence. Drops of physiological solution (EUROSPITAL, Trieste, Italy) were sprinkled to keep the interface between the films and the biological tissue wet through the entire procedure. Laser light was delivered as one pulse per spot. Relevant parameters were set as output power of 1.0 W and duration of 200 ms, which were established after an exploratory search with the tendons. The setup implemented in this model was one piece of tendon bonded to one piece of film with a total of 20 spots. In the case of the dermis, instead, two pieces of biological tissue were bonded to the same piece of film so as to mimic the treatment of a surgical incision with 20 spots per side of the simulated wound. In both cases, the distance from spot to spot was always larger than the diameter of the fiber optic tip in order to avoid their juxtaposition. We emphasize that the experimental setup was used as a quantitative assessment of the strength of the adhesion rather than to deploy a real clinical protocol, which would probably require a higher density of spots.

After welding, samples were prepared for tensile testing. In the case of the tendons, the distal grip was fixed to the biological tissue and the proximal one was fixed to the film. In the case of the skin, instead, each of the two grips were fixed to the opposite sides of the simulated wound. With this setup, the same system used for tensile testing of the films now actually imparts a quasi shear stress, because the applied load is, at least initially, parallel to the welded interface. The stress was computed as the ratio of the applied load divided by 20× the surface area of each spot under the conservative hypothesis that the distribution of the pull is even across all welds, which probably overestimates the actual value, especially as any of them start failing during the run.

The numbers of samples implemented for tensile testing were 15 for the tendons and 5 for the skin.

## 3. Results

### 3.1. Visual Appearance of the Films

**Figure 1:**
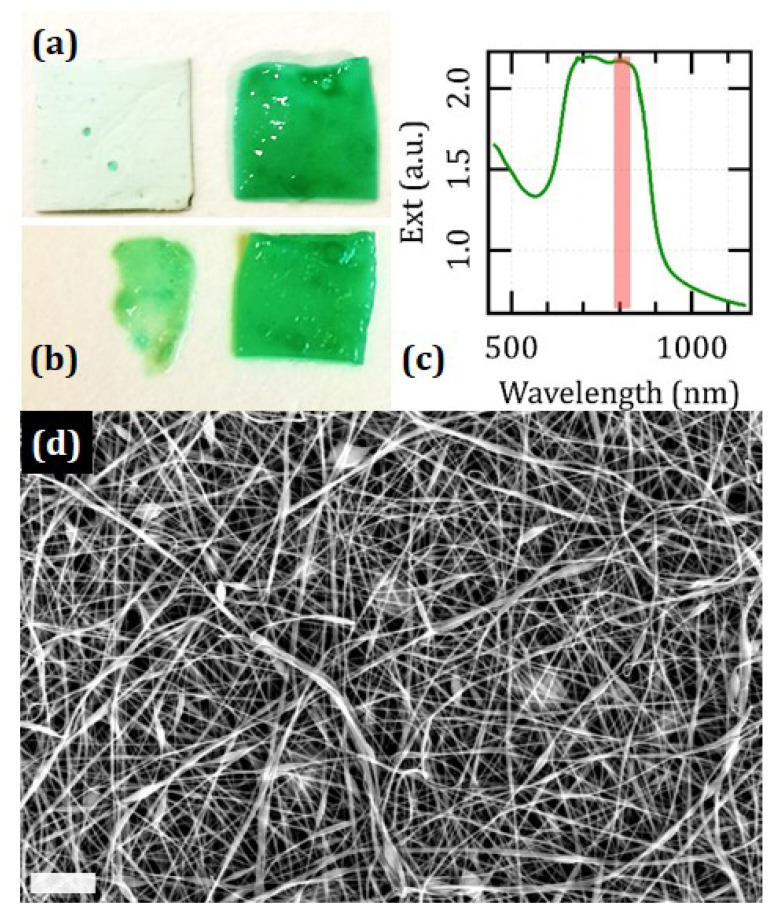
Visual appearance and ultrastructure of the films: Photographs of specimens of the films of a size around 1 cm${}^{2}$ (**a**) before (**left**) and after (**right**) hydration in a physiological buffer and (**b**) upon hydration before (**left**) and after (**right**) cross-linking. The green spots in the dry sample are usual defects originating from occasional instabilities and dripping in the process of electrospinning. Note that the sample before cross-linking undergoes partial dissolution upon soaking in physiological buffer for 30 min, and so it is only partially recovered after hydration. (**c**) Spectrum of optical extinction of a cross-linked sample after a slight hydration taken with a V-770 device from Jasco (Tokyo, Japan). The pink line represents the optical emission of the diode laser used for welding. (**d**) Representative SEM micrograph of a sample before hydration. The scale bar corresponds to 5 μm.

Figure 1 displays the macroscopic and ultrastructural appearance of the films. We obtained thin sheets that seem as intangible and opaque as tissue paper but readily become bright green upon contact with a moist substrate and hydration. The process of cross-linking proves to not affect the macroscopic appearance of the films but rather their compatibility with a wet environment. Unlike the as-spun mats that undergo progressive dissolution in a physiological buffer over a timescale of a few min, the cross-linked samples swell by about two times but remain intact for several days. We considered whether the process of cross-linking may undermine the optical features of pristine ICG. Before hydration, too much scattering prevents the acquisition of spectra of optical extinction, which therefore must exceed the range of 10${}^{4}$ cm${}^{-1}$ until at least 1.2 μm. However, a slight hydration is enough to bring out the typical features of a high concentration of aqueous ICG. In particular, we found a distinct contribution around 800 nm that we attribute to a dissipative process of optical absorbance, which displays a density around 10${}^{3}$ cm${}^{-1}$. The extent of the initial opacity points to a high degree of porosity of the as-synthesized mats. Indeed, their ultrastructural analysis reveals a dense maze of fibrils of a typical diameter of (140 ± 40) nm, which occasionally happen to assemble in small bundles over lengths of tens or hundreds of microns.

In the Supplementary Material, we provide additional data obtained by Fourier-transform infrared spectroscopy (FTIR) [45,46,47] and Powder X-ray Diffraction (PXRD) [48] that corroborate the chemical composition of the cross-linked films and suggest the formation of an intimate blend of PVA and chitosan as opposed to separate microphases.

### 3.2. Mechanical Strength of the Films under Operative Conditions

**Figure 2:**
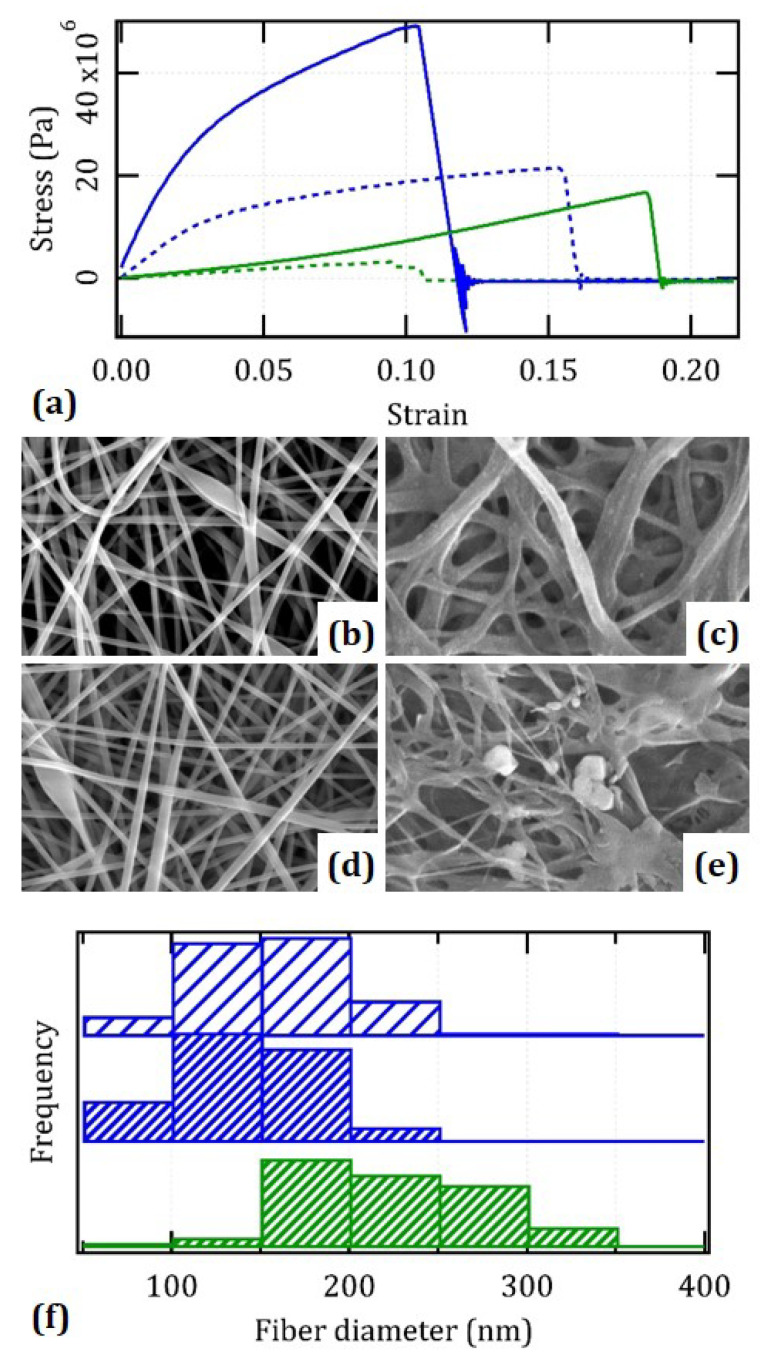
Tensile testing of the films and its microscopic interpretation: (**a**) representative stress–strain curves for the samples before (blue) and after (green) hydration in physiological buffer both before (dotted) and after (continuous) cross-linking. All curves were drawn from the nominal thickness of the films in air, i.e., 11 μm. The 10 μm × 6 μm SEM micrographs of cross-linked (**b**,**c**) and as-spun (**d**,**e**) samples both before (**b**,**d**) and after (**c**,**e**) hydration. (**f**) Relevant distributions of fiber diameters computed both before (blue) and after (green) hydration, and before (light) and after (dark) cross-linking. Note that it was impossible to assess the fiber diameters in the case of the as-spun samples after hydration due to their largely compromised morphology.

We tested the mechanical properties of the films in order to assess their potential for integration in the skin. Figure 2 summarizes all results. After hydrating as a means to simulate the operative conditions, we found the typical superlinear behavior of elastomeric materials that is peculiar of the skin [18,19,20], and originates from the alignment and mobility of the underlying fibers. A typical value for the initial modulus in the limit of no strain is E = 51 MPa ± 20%, which resembles that of the dermis, and the ultimate tensile strength is around UTS = 17 MPa ± 30%. We note that before hydration, films are much stiffer and brittler and display E = 1.1 GPa ± 20% and UTS = 47 MPa ± 30%. Their stress–strain curve in dry conditions shows the typical concavity of ductile materials with a large plastic range after Hooke’s regime, a yield point below about 2% and strength around 20 MPa. The cross-linking procedure is critical for the overall feasibility of these materials. Without cross-linking and upon hydration, films are rather weak and exhibit E = 30 MPa ± 20% and UTS = 3.1 MPa ± 30% only. The relevant values in dry conditions are E = 420 MPa ± 20% and UTS = 21 MPa ± 30%, and the concavity is still there, i.e., it is clearly not a unique consequence of cross-linking.

In an attempt to address the correlation between the mechanical and morphological data, we undertook an ultrastructural inspection of all samples. It turns out that the fibers undergo swelling during hydration from the initial value of (140 ± 40) nm to (220 ± 60) nm, yet while maintaining their integrity. On visual inspection, swelling occurs within a few min and then stabilizes for at least several days. By comparison with the as-spun mats, we note that cross-linking induces mild shrinkage by about 13% from an interim value of (160 ± 50) nm. Without cross-linking, fibers are incompatible with hydration and lose their integrity within about 30 min. The relevant SEM micrographs display a mixture of signs of thinning and deliquescence, which evolve toward complete dissolution within hours.

### 3.3. Feasibility for Laser Bonding to Rabbit Tendons and Human Dermis

**Figure 3:**
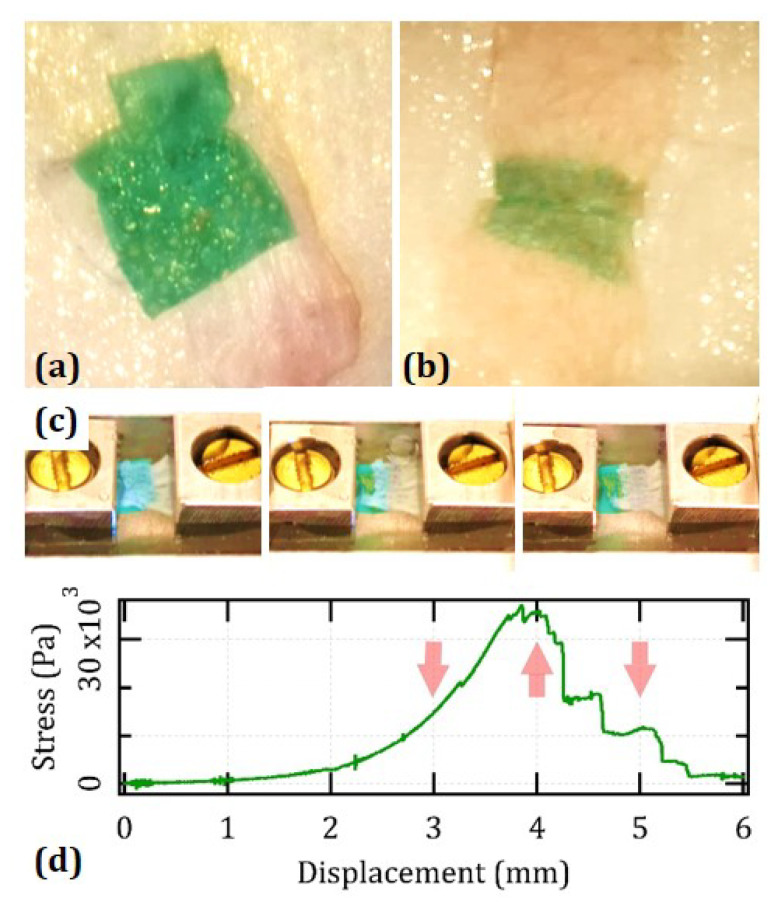
Laser bonding to biological tissue and its mechanical strength: Photographs of films welded to a segment of rabbit tendon (**a**) and a simulated wound in human skin (**b**). The irradiated spots look clearer than their neighborhood probably due to the photobleaching of ICG and an ultrastructural modification of the mats. (**c**) Snapshots taken during a measuring run in the case of shear testing of the adhesion between a sample and a piece of rabbit tendon. (**d**) Relevant stress–strain curve calculated by normalizing by the total nominal surface area of the 20 spots. The arrows roughly correspond to the snapshots reported in panel (**c**) and illustrate that the breakdown of the seam occurs by the progressive failure of individual spots one by one or in small subgroups.

We verified the feasibility of the films for tissue bonding. Figure 3 provides an overview over the appearance of the samples welded both on rabbit tendons and pieces of human dermis. In the case of the tendons, the experimental setup was specifically designed for mechanical testing. In that of the dermis, it simulates the treatment of surgical incisions. In both models, individual pulses of a fluence around 280 J × cm${}^{-2}$ and a duration of 200 ms were suitable to trigger an adhesive effect of a strength that was sufficient to withstand extensive manipulation, similar to our previous generation of hydrogels made of pure chitosan [9,10,11,14]. Visual signs of thermal damage were minimal and, under a surgical microscope, were always seen to lie well within the welds or their immediate proximities [44].

We tested the strength of the welds against a quasi-tangential load. Panels 3 (c) and (d) display a representative example in the case of a segment of rabbit tendon. In both models, the stress–strain curves exhibit a broad envelope and a series of irregular crests that correspond to the progressive failure of individual welds. The global maximum rarely exceeds the first two or three crests. We calculated an effective value of the strength of the welds by considering the total surface area of the 20 spots. In the rabbit tendons, we found avg UTS = (28 ± 6) kPa. In human dermis, it was about 20% lower, i.e., avg UTS = (22 ± 9) kPa. Note that, however, in the case of the skin, the experimental setup is such that the measured UTS always relates to the weakest of the two sides of the simulated wound, where, for instance, the limiting factor may be the incidence of faulty spots. Some lack of reproducibility from spot to spot may originate from factors that may be inevitable such as the distinctive heterogeneity of the connective tissue or more controllable, such as the density of defects in the electrospun films (see panel 1 (a)). A single broken spot out of 20 or, in the case of the skin, out of 40 is sufficient to downgrade the overall performance of the entire procedure. In addition, remember that the laser parameters were optimized with the tendons and not the skin, which was available in more limited quantity.

## 4. Discussion

By using films made entirely of FDA-approved and natural components, this work aims to prioritize the dimension of biocompatibility and potential for translational development. PVA is already used in a variety of medical applications because of its low toxicity [49] and low tendency to interact with proteins. Specific uses include, for instance, its integration in cartilage replacements [50], contact lenses and eye drops [51], as well as wound dressings [4,5]. Chitosan is a polysaccharide obtained from the deacetylation of chitin, which is a natural component found in the exoskeleton of crustaceans and the cell walls of mushrooms. It was approved for integration in wound dressings in the USA back in 2003 [52,53]. It provides hemostatic activity and may also inhibit the proliferation of bacteria and fungi [2]. ICG is a cyanine dye that was approved for medical diagnostics in the USA as early as 1959 [54]. It is used in a plethora of medical contexts that include the determination of cardiac output, hepatic function, liver and gastric blood flow, and for ophthalmic angiography [55]. In this context, the use of GLA may give rise to legitimate reservations. We found that cross-linking is a critical step to gain compatibility with a moist environment such as that met in operative conditions and mechanical strength.

GLA is an obvious option to cross-link chitosan, due to its high density of free amines, as well as PVA, owing to its hydroxyl moieties [56,57], and their blends [29,30,32]. Our results are comparable with those reported by Yao et al. [58], where mats made of pure PVA were stabilized by physical cross-linking upon soaking in ethanol to remove water and trigger inter-molecular interactions, as we too did in our previous work [13]. Their mats achieved E = 1.3 GPa from an initial value of 93 MPa prior to cross-linking and E = 6.7 MPa after immersion in water, which is only about 0.5% of 1.3 GPa. In comparison, our respective values are about 1.1 GPa after chemical cross-linking, 420 MPa as spun, and 51 MPa upon hydration, or almost 5% of 1.1 GPa. The greater stability of our films in water therefore seems to originate from an interplay of the chemical effect of GLA and the presence of inter-molecular interactions between PVA and the insoluble polysaccharide [13]. Indeed, the FTIR and PXRD data reported in the Supplementary Material collectively corroborate the notion that PVA and chitosan form an intimate blend without substantial phase separation, where chemical cross-linking is key to retard the loss of the water-soluble component. Here, we refrained from soaking in ethanol or treating with physical agents such as heat [59] in order to avoid any release or deterioration of ICG. Moreover, in our previous work [60] and in the Supplementary Material, we show that cross-linking with GLA does not raise substantial issues in terms of cytotoxicity, which is also consistent with the findings by Li et al. [29]. Here, we focused on the merit of GLA as a solution to prevent the individual fibers from dissolving, although it allows them to hydrate and swell in a physiological buffer.

Hydrated samples display the optical behavior of concentrated ICG in aqueous buffer [61], which is consistent with the observation that the initial concentration of 0.31% corresponds to around 4.8 mmol/L in the solid mat. The peak of optical absorbance around 10${}^{3}$ cm${}^{-1}$ therefore corresponds to a contribution of ICG in the order of 10${}^{5}$ M${}^{-1}$× cm${}^{-1}$, which points to a substantial integrity of the dye [61]. Indeed, no change in the optical properties was ever noticed over at least 6 months under ambient conditions in the dark after spinning and cross-linking. Similar results were found for the integration of ICG in pure chitosan [11,14].

Before hydration, films display the mechanical behavior of ductile materials, as if the fibers were not free to realign and slide according to the applied strain. We hypothesize that this behavior may correlate with the extent of inter-fibrillar bonds [62]. However, such a reticulation is not a unique outcome of cross-linking alone, because the same trend is already there in the stress–strain curve of the as-spun samples. Instead, after hydration, films display the mechanical behavior of elastomeric materials, as if the inter-fibrillar bonds weakened in physiological buffer and viscosity could take place. In the meantime, the hydrated samples gain substantial extensibility, which exceeds about 15% before rupture. The cross-linked films exhibit a mechanical strength that is close to or generally higher than that of similar materials [63]. For instance, Kohsari et al. [64] reported a value for UTS of 8.94 MPa and E in the order of 100 MPa for a blend containing as much as 75% chitosan and 25% polyethylene oxide and treated with a vapor of GLA for just 15 min, where both parameters increase upon the addition of silver nanospheres. In the case of pure PVA, Jeong et al. [65] found UTS = 5.8 MPa and E = 175 MPa, and both values fluctuated upon blending with carbon nanotubes. Their graphs suggest an ultimate extensibility of pure or doped PVA in the plastic regime in excess of 100%. Instead, Destaye et al. [57] reported UTS = 4.4 MPa, which almost tripled upon exposure to a vapor of GLA until 12.7 MPa. More recently, Koosha et al. [66] reported a blend containing 12% chitosan and 88% PVA featuring the behavior of ductile materials with UTS = 19.7 MPa, E in the order of 400 MPa and an extensibility of 16.2%, which resembles our data for the as-spun mats in air. However, at variance with the effect of GLA that we observed here, they found that cross-linking with glyoxal essentially counterintuitively halved both their values for UTS and E. Instead, the addition of halloysite nanotubes imparted a substantial reinforcement to the hybrid samples. In our case, we hypothesize that ICG may concur to consolidate the fibrous scaffold by interacting with chitosan [67].

In any case, the overall strength of the films proves to be compatible with laser bonding. Their consistency and conformability are ideal for manipulation and application. After cutting in arbitrary shapes with surgical scissors and laying in place, films closely cohere to the underlying tissue at first contact, such as tissue paper. In contrast, our former generation of chitosan hydrogels [9,10,11,14] was rigid and often tended to roll up according to the stress accumulated during their preparation. The thickness of the films is such that they absorb >90% of the laser light delivered at 810 nm, and that their distal interface to the biological tissue becomes as hot as their proximal side during irradiation, heat diffusing over a distance in the order of 170 μm within the duration of the optical pulse, which is about 10 times larger than the size of the swollen samples. Therefore, the system is as efficient as possible in terms of photothermal conversion. Although the bio-active component is a minority ingredient, with a fraction of chitosan around 16%, welding was successful as well.

The mechanical strength of the welds found in this work is compatible with that described in our previous papers [9,10,11,13,14]. As a relevant benchmark, Lauto et al. [68,69] also found a comparable strength around 15 kPa by bonding chitosan hydrogels containing either ICG or rose bengal to sheep intestine. Likewise, Gobin et al. [70] obtained a similar value around 30 kPa by using an albumin paste doped with gold nanoshells both in the case of chicken muscle and rat skin. Instead, a higher value of 120 kPa was reported by Schönfeld et al. [71] by implementing a 300 μm-thick albuminated mat of 1.2 μm-thick electrospun fibers made of polycaprolactone and ICG in the case of rabbit aorta irradiated with a dedicated diffusor fiber from an intraluminal balloon catheter. However, their result was worked out by dividing the ultimate load by the cross-sectional area of the anastomosed blood vessel rather than the surface area of the working weld, which is an alternative definition of mechanical strength based on effective anatomical data.

In conclusion, we have shown that a reformulation of our materials into about 10 μm-thick mats of bioactive nanofibers of an average diameter of (140 ± 40) nm resembling the ultrastructure of human dermis is suitable to achieve an overall consistency and mechanical features compatible with integration in the skin. By using FDA-approved and natural components such as PVA, chitosan and ICG and a mild treatment with GLA, we obtained films that are ideally conformable and compatible with a moist environment such as that met in operative conditions for laser bonding. In particular, we found an elastomeric behavior with E = 51 MPa ± 20% in the limit of no strain, UTS = 17 MPa ± 30% and extensibility exceeding 15%, whereas the corresponding values for the skin respectively fall in the ranges of about 20 to 40 MPa, 10 to 30 MPa and 40 to 60%. While there still remains room for improvement, we believe that electrospinning is a remarkable choice to replicate the relationship between ultrastructure and mechanical function of biological tissue. Upon optical irradiation, the films were suitable to weld to human dermis with a strength of (22 ± 9) kPa, which is similar to the value found by Gobin et al. [70] in the related case of rat skin by employing an albumin paste. This observation suggests that the peculiar consistency of the electrospun mats allows their conformance to the underlying bio-tissue to an extent comparable to a viscous solution while retaining the practical advantages of usual dressings.

In the future, we will address the standardization of our protocols both for the production of electrospun materials and the optical procedure [6], in order to minimize, for instance, the incidence of faulty spots. The demonstration of the ability of our films to outperform current methods in use in wound dressing in terms of medical outcome will eventually require testing in vivo [8,11,42]. However, prior to that, we will direct our attention to the enhancement of the feasibility of the electrospun scaffolds for cell repopulation. We anticipate that the present formulation seems to be less than ideal as a substrate for representative cell types, such as human fibroblasts. The emergence of this issue is common to previous cases of blends of chitosan and PVA [66,72] or other materials predominantly based on PVA [73,74,75], which also suggest replicable directions for improvement, such as the increase of the percent of chitosan [72] or other polysaccharides [74], or the incorporation of various components such as aluminosilicates [66], relevant growth factors [73] or scleroproteins [75]. We will also address the possibility to load and controllably release drugs and other factors, as we have done in our previous work on chitosan hydrogels [12]. Our long-term objective is the demonstration of a unique platform that may reconcile the functions of wound dressing, controlled drug release and tissue engineering.

## Data Availability

Data are available from the authors.

## Supplementary Materials

The following are available online at https://www.mdpi.com/article/10.3390/nano12091613/s1, Figure S1: Fourier-transform infrared spectroscopy of the electrospun films, Figure S2: X-ray diffraction of the cross-linked electrospun films, Figure S3: Cytotoxicity of the electrospun films upon cross-linking.

## Sample Availability

Samples of the composite mats are available from the authors.

## References

[1] Rossi F., Matteini P., Ratto F., Menabuoni L., Lenzetti I., Pini R. (2008). Laser tissue welding in ophthalmic surgery. J. Biophoton..

[2] Jayakumar R., Prabaharan M., Sudheesh Kumar P.T., Nair S.V., Tamura H. (2011). Biomaterials based on chitin and chitosan in wound dressing applications. Biotechnol. Adv..

[3] Matteini P., Ratto F., Rossi F., Pini R. (2012). Emerging concepts of laser-activated nanoparticles for tissue bonding. J. Biomed. Opt..

[4] Kamoun E.A., Chen X., Eldin M.S.M., Kenawy E.R.S. (2015). Crosslinked poly (vinyl alcohol) hydrogels for wound dressing applications: A review of remarkably blended polymers. Arab. J. Chem..

[5] Kamoun E.A., Kenawy E.S., Chen X. (2017). A review on polymeric hydrogel membranes for wound dressing applications: PVA-based hydrogel dressings. J. Adv. Res..

[6] Basov S., Milstein A., Sulimani E., Platkov M., Peretz E., Rattunde M., Wagner J., Netz U., Katzir A., Nisky I. (2018). Robot-assisted laser tissue soldering system. Biomed. Opt. Express..

[7] Stoica A.E., Chircov C., Grumezescu A.M. (2020). Nanomaterials for Wound Dressings: An Up-to-Date Overview. Molecules.

[8] Matteini P., Ratto F., Rossi F., Rossi G., Esposito G., Puca A., Albanese A., Maira G., Pini R. (2010). In vivo carotid artery closure by laser activation of hyaluronan-embedded gold nanorods. J. Biomed. Opt..

[9] Matteini P., Ratto F., Rossi F., Centi S., Dei L., Pini R. (2010). Chitosan films doped with gold nanorods as laser-activatable hybrid bioadhesives. Adv. Mater..

[10] Matteini P., Ratto F., Rossi F., de Angelis M., Cavigli L., Pini R. (2012). Hybrid nanocomposite films for laser-activated tissue bonding. J. Biophotonics.

[11] Esposito G., Rossi F., Matteini P., Scerrati A., Puca A., Albanese A., Rossi G., Ratto F., Maira G., Pini R. (2013). In vivo laser assisted microvascular repair and end-to-end anastomosis by means of indocyanine green-infused chitosan patches: A pilot study. Lasers Surg. Med..

[12] Matteini P., Tatini F., Luconi L., Ratto F., Rossi F., Giambastiani G., Pini R. (2013). Photothermally Activated Hybrid Films for Quantitative Confined Release of Chemical Species. Angew. Chem. Int. Ed..

[13] Milanesi A., Magni G., Centi S., Schifino G., Aluigi A., Khlebtsov B.N., Cavigli L., Barucci A., Khlebtsov N.G., Ratto F. (2020). Optically activated and interrogated plasmonic hydrogels for applications in wound healing. J. Biophotonics.

[14] Rossi F., Magni G., Colasanti R., Banchelli M., Iacoangeli M., Carrassi E., Aiudi D., Di Rienzo A., Giannoni L., Pieri L. (2021). Characterization and Ex Vivo Application of Indocyanine Green Chitosan Patches in Dura Mater Laser Bonding. Polymers.

[15] Khlebtsov N., Dykman L. (2011). Biodistribution and toxicity of engineered gold nanoparticles: A review of in vitro and in vivo studies. Chem. Soc. Rev..

[16] Brown T.M., Krishnamurthy K. (2022). Histology, Dermis.

[17] Mercatelli R., Mattana S., Capozzoli L., Ratto F., Rossi F., Pini R., Fioretto D., Pavone F.S., Caponi S., Cicchi R. (2019). Morpho-mechanics of human collagen superstructures revealed by all-optical correlative micro-spectroscopies. Commun. Biol..

[18] Silver F.H., Freeman J.W., DeVore D. (2001). Viscoelastic properties of human skin and processed dermis. Skin Res. Technol..

[19] Yang W., Sherman V.R., Gludovatz B., Schaible E., Stewart P., Ritchie R.O., Meyers M.A. (2015). On the tear resistance of skin. Nat. Commun..

[20] Joodaki H., Panzer M.B. (2018). Skin mechanical properties and modeling: A review. Proc. Inst. Mech. Eng. Part H.

[21] Aziz J., Shezali H., Radzi Z., Yahya N.A., Abu Kassim N.H., Czernuszka J., Rahman M.T. (2016). Molecular Mechanisms of Stress-Responsive Changes in Collagen and Elastin Networks in Skin. Skin Pharmacol. Physiol..

[22] Kalra A., Lowe A., Al-Jumaily A.M. (2016). Mechanical Behaviour of Skin: A Review. J. Mater. Sci. Eng..

[23] Lei Z., Wu P. (2018). A supramolecular biomimetic skin combining a wide spectrum of mechanical properties and multiple sensory capabilities. Nat. Commun..

[24] Ohkawa K., Cha D., Kim H., Nishida A., Yamamoto H. (2004). Electrospinning of Chitosan. Macromol. Rapid Commun..

[25] Jia Y.T., Gong J., Gu X.H., Kim H.Y., Dong J., Shen X.Y. (2007). Fabrication and characterization of poly (vinyl alcohol)/chitosan blend nanofibers produced by electrospinning method. Carbohydr. Polym..

[26] Zhang Y., Huang X., Duan B., Wu L., Li S., Yuan X. (2007). Preparation of electrospun chitosan/poly(vinyl alcohol) membranes. Colloid Polym. Sci..

[27] Aluigi A., Vineis C., Varesano A., Mazzuchetti G., Ferrero F., Tonin C. (2008). Structure and properties of keratin/PEO blend nanofibres. Eur. Polym. J..

[28] Hang A.T., Tae B., Park J.S. (2010). Non-woven mats of poly(vinyl alcohol)/chitosan blends containing silver nanoparticles: Fabrication and characterization. Carbohydr. Polym..

[29] Li C., Fu R., Yu C., Li Z., Guan H., Hu D., Zhao D., Lu L. (2013). Silver nanoparticle/chitosan oligosaccharide/poly(vinyl alcohol) nanofibers as wound dressings: A preclinical study. Int. J. Nanomed..

[30] Abdelgawad A.M., Hudson S.M., Rojas O.J. (2014). Antimicrobial wound dressing nanofiber mats from multicomponent (chitosan/silver-NPs/polyvinyl alcohol) systems. Carbohydr. Polym..

[31] Koosha M., Mirzadeh H. (2015). Electrospinning, mechanical properties, and cell behavior study of chitosan/PVA nanofibers. J. Biomed. Mater. Res. A.

[32] Wang M., Roy A.K., Webster T.J. (2017). Development of Chitosan/Poly(Vinyl Alcohol) Electrospun Nanofibers for Infection Related Wound Healing. Front. Physiol..

[33] Xue J., Wu T., Dai Y., Xia Y. (2019). Electrospinning and Electrospun Nanofibers: Methods, Materials, and Applications. Chem. Rev..

[34] Goh Y.F., Shakir I., Hussain R. (2013). Electrospun fibers for tissue engineering, drug delivery, and wound dressing. J. Mater. Sci..

[35] Liu Y., Li T., Han Y., Li F., Liu Y. (2021). Recent development of electrospun wound dressing. Curr. Opin. Biomed. Eng..

[36] Yang W., Fortunati E., Bertoglio F., Owczarek J.S., Bruni G., Kozanecki M., Kenny J.M., Torre L., Visai L., Puglia D. (2018). Polyvinyl alcohol/chitosan hydrogels with enhanced antioxidant and antibacterial properties induced by lignin nanoparticles. Carbohydr. Polym..

[37] Abbas M., Hussain T., Arshad M., Ansari A.R., Irshad A., Nisar J., Hussain F., Masood N., Nazir A., Iqbal M. (2019). Wound healing potential of curcumin cross-linked chitosan/polyvinyl alcohol. Int. J. Biol. Macromol..

[38] Zou P., Lee W.H., Gao Z., Qin D., Wang Y., Liu J., Sun T., Gao Y. (2020). Wound dressing from polyvinyl alcohol/chitosan electrospun fiber membrane loaded with OH-CATH30 nanoparticles. Carbohydr. Polym..

[39] Mohanty S., Larsen L.B., Trifol J., Szabo P., Burri H.V., Canali C., Dufva M., Emnéus J., Wolff A. (2015). Fabrication of scalable and structured tissue engineering scaffolds using water dissolvable sacrificial 3D printed moulds. Mater. Sci. Eng. C Mater. Biol..

[40] Tocchio A., Tamplenizza M., Martello F., Gerges I., Rossi E., Argentiere S., Rodighiero S., Zhao W., Milani P., Lenardi C. (2015). Versatile fabrication of vascularizable scaffolds for large tissue engineering in bioreactor. Biomaterials.

[41] Giuri D., Barbalinardo M., Sotgiu G., Zamboni R., Nocchetti M., Donnadio A., Corticelli F., Valle F., Gennari C.G.M., Selmin F. (2019). Nano-hybrid electrospun non-woven mats made of wool keratin and hydrotalcites as potential bio-active wound dressings. Nanoscale.

[42] Rossi F., Pini R., Menabuoni L., Mencucci R., Menchini U., Ambrosini S., Vannelli G. (2005). Experimental study on the healing process following laser welding of the cornea. J. Biomed. Opt..

[43] Pini R., Rossi F., Menabuoni L., Lenzetti I., Yoo S., Parel J.M. (2008). A new technique for the closure of the lens capsule by laser welding. Ophthalmic Surg. Lasers Imaging.

[44] Ratto F., Matteini P., Rossi F., Menabuoni L., Tiwari N., Kulkarni S.K., Pini R. (2009). Photothermal effects in connective tissues mediated by laser-activated gold nanorods. Nanomedicine.

[45] Rosli N., Yahya W.Z.N., Wirzal M.D.H. (2022). Crosslinked chitosan/poly(vinyl alcohol) nanofibers functionalized by ionic liquid for heavy metal ions removal. Int. J. Biol. Macromol..

[46] Jung B., Anvari B. (2012). Synthesis and characterization of bovine serum albumin-coated nanocapsules loaded with indocyanine green as potential multifunctional nanoconstructs. Biotechnol. Prog..

[47] Fernandes Queiroz M., Melo K.R., Sabry D.A., Sassaki G.L., Rocha H.A. (2014). Does the use of chitosan contribute to oxalate kidney stone formation?. Mar. Drugs.

[48] Holzwarth U., Gibson N. (2011). The Scherrer equation versus the ‘Debye–Scherrer equation’. Nat. Nanotechnol..

[49] DeMerlis C.C., Schoneker D.R. (2003). Review of the oral toxicity of polyvinyl alcohol (PVA). Food Chem. Toxicol..

[50] Baker M.I., Walsh S.P., Schwartz Z., Boyan B.D. (2005). A review of polyvinyl alcohol and its uses in cartilage and orthopedic applications. J. Biomed. Mater. Res. B Appl. Biomater..

[51] Franco P., De Marco I. (2021). Contact Lenses as Ophthalmic Drug Delivery Systems: A Review. Polymers.

[52] Zhang Y.J., Gao B., Liu X.W. (2015). Topical and effective hemostatic medicines in the battlefield. Int. J. Clin. Exp. Med..

[53] Singh R., Shitiz K., Singh A. (2017). Chitin and chitosan: Biopolymers for wound management. Int. Wound J..

[54] Bates A.S., Patel V.R. (2016). Applications of indocyanine green in robotic urology. J. Robot Surg..

[55] Alander J.T., Kaartinen I., Laakso A., Pätilä T., Spillmann T., Tuchin V.V., Venermo M., Välisuo P. (2012). A review of indocyanine green fluorescent imaging in surgery. Int. J. Biomed. Imaging.

[56] Mansur H.S., Sadahira C.M., Souza A.N., Mansur A.A.P. (2008). FTIR spectroscopy characterization of poly (vinyl alcohol) hydrogel with different hydrolysis degree and chemically crosslinked with glutaraldehyde. Mater. Sci. Eng. C.

[57] Destaye A.G., Lin C.K., Lee C.K. (2013). Glutaraldehyde Vapor Cross-linked Nanofibrous PVA Mat with in Situ Formed Silver Nanoparticles. ACS Appl. Mater. Interfaces.

[58] Yao L., Haas T.W., Guiseppi-Elie A., Bowlin G.L., Simpson D.G., Wnek G.E. (2003). Electrospinning and Stabilization of Fully Hydrolyzed Poly(Vinyl Alcohol) Fibers. Chem. Mater..

[59] Göksen G., Fabra M.J., Pérez Cataluña A., Ekiz H.I., Sánchez Moragas G., López Rubio A. (2021). Biodegradable active food packaging structures based on hybrid cross-linked electrospun polyvinyl alcohol fibers containing essential oils and their application in the preservation of chicken breast fillets. Food Packag. Shelf Life.

[60] Ratto F., Milanesi A., Magni G., Centi S., Schifino G., Aluigi A., Khlebtsov B.N., Cavigli L., Barucci A., Matteini P. (2021). Electrospinnable composites for laser-activated tissue bonding and wound monitoring. Proc. SPIE.

[61] Landsman M.L.J., Kwant G., Mook G.A., Zijlstra W.G. (1976). Light-absorbing properties, stability, and spectral stabilization of indocyanine green. J. Appl. Physiol..

[62] Arola D., Ghods S., Son C., Murcia S., Ossa E.A. (2019). Interfibril hydrogen bonding improves the strain-rate response of natural armour. J. R. Soc. Interface.

[63] Rashid T.U., Gorga R.E., Krause W.E. (2021). Mechanical Properties of Electrospun Fibers—A Critical Review. Adv. Eng. Mater..

[64] Kohsari I., Shariatinia Z., Pourmortazavi S.M. (2016). Antibacterial electrospun chitosan-polyethylene oxide nanocomposite mats containing bioactive silver nanoparticles. Carbohydr. Polym..

[65] Jeong J.S., Moon J.S., Jeon S.Y., Park J.H., Alegaonkar P.S., Yoo J.B. (2007). Mechanical properties of electrospun PVA/MWNTs composite nanofibers. Thin Solid Film..

[66] Koosha M., Raoufi M., Moravvej H. (2019). One-pot reactive electrospinning of chitosan/PVA hydrogel nanofibers reinforced by halloysite nanotubes with enhanced fibroblast cell attachment for skin tissue regeneration. Colloids Surf. B Biointerfaces.

[67] Porcu E.P., Salis A., Gavini E., Rassu G., Maestri M., Giunchedi P. (2016). Indocyanine green delivery systems for tumour detection and treatments. Biotechnol. Adv..

[68] Lauto A., Stoodley M., Marcel H., Avolio A., Sarris M., McKenzie G., Sampson D.D., Foster L.J. (2007). In vitro and in vivo tissue repair with laser-activated chitosan adhesive. Lasers Surg. Med..

[69] Lauto A., Stoodley M., Barton M., Morley J.W., Mahns D.A., Longo L., Mawad D. (2012). Fabrication and application of rose bengal-chitosan films in laser tissue repair. J. Vis. Exp..

[70] Gobin A.M., O’Neal D.P., Watkins D.M., Halas N.J., Drezek R.A., West J.L. (2005). Near infrared laser-tissue welding using nanoshells as an exogenous absorber. Lasers Surg. Med..

[71] Schönfeld A., Kabra Z.M., Constantinescu M., Bosshardt D., Stoffel M.H., Peters K., Frenz M. (2017). Binding of indocyanine green in polycaprolactone fibers using blend electrospinning for in vivo laser-assisted vascular anastomosis. Lasers Surg. Med..

[72] Koyano T., Minoura N., Nagura M., Kobayashi K. (1998). Attachment and growth of cultured fibroblast cells on PVA/chitosan-blended hydrogels. J. Biomed. Mater. Res..

[73] Asiri A., Saidin S., Sani M.H., Al-Ashwal R.H. (2021). Epidermal and fibroblast growth factors incorporated polyvinyl alcohol electrospun nanofibers as biological dressing scaffold. Sci. Rep..

[74] Kraskouski A., Hileuskaya K., Kulikouskaya V., Kabanava V., Agabekov V., Pinchuk S., Vasilevich I., Volotovski I., Kuznetsova T., Lapitskaya V. (2021). Polyvinyl alcohol and pectin blended films: Preparation, characterization, and mesenchymal stem cells attachment. J. Biomed. Mater. Res. A..

[75] Mohamed J.M.M., Alqahtani A., Al Fatease A., Alqahtani T., Khan B.A., Ashmitha B., Vijaya R. (2021). Human Hair Keratin Composite Scaffold: Characterisation and Biocompatibility Study on NIH 3T3 Fibroblast Cells. Pharmaceuticals.

