# Supplementary material for: Cyanine-Doped Nanofiber Mats for Laser Tissue Bonding
Source: Nanomaterials (Basel). 2022 May 9;12(9):1613. doi: 10.3390/nano12091613 (PMC9105542; doi:10.3390/nano12091613)
Supplement: Supplementary file 1 [file nanomaterials-12-01613-s001.zip › nanomaterials-1684404-supplementary.pdf]

Supplementary Material

Cyanine-Doped Nanofiber Mats for Laser Tissue Bonding

Fulvio Ratto [1], Giada Magni [1], Annalisa Aluigi [2,†], Marta Giannelli [2], Sonia Centi [1], Paolo Matteini [1], Werner Oberhauser [3], Roberto Pini [1] and Francesca Rossi [1]

1 Istituto di Fisica Applicata "Nello Carrara", Consiglio Nazionale delle Ricerche, via Madonna del Piano 10, 50019 Sesto Fiorentino (FI), Italy 2 Istituto per la Sintesi Organica e la Fotoreattività, Consiglio Nazionale delle Ricerche, via P. Gobetti 101, 40129 Bologna, Italy 3 Istituto di Chimica dei Composti Organometallici, Consiglio Nazionale delle Ricerche, via Madonna del Piano 10, 50019 Sesto Fiorentino (FI), Italy

* Correspondence: f.ratto@ifac.cnr.it (F.Ra); f.rossi@ifac.cnr.it (F.Ro)

† Current address: Dipartimento di Scienze Biomolecolari, Università di Urbino Carlo Bo, Piazza Rinascimento 6, 61029 Urbino (PU), Italy

Fourier-transform infrared spectroscopy of the electrospun films

Fourier-transform infrared (FTIR) spectra were acquired with a Vertex 70 interferometer from Bruker (Billerica, MA, USA) equipped with a diamond crystal single reflection Platinum ATR accessory, in the region from 4000 to 600 cm^{-1}, with 100 scans and a resolution of 4 cm^{-1}.

As shown in Figure S1, the FTIR-ATR spectra of all samples show the characteristic adsorption peaks of chitosan, polyvinyl alcohol (PVA) and indocyanine green (ICG). In particular, the broad adsorption band centered around 3300 cm^{-1} contains the contributions of the OH stretching vibration of chitosan and PVA, as well as the NH stretching vibration of chitosan [1]. The adsorption peaks in the region from 3000 to 2800 cm^{-1} are typical of the CH asymmetric and symmetric vibrations of the CH_2 groups of chitosan, PVA as well as ICG, while the feature at 1424 cm^{-1} relates to the CH_3 asymmetric bending vibration of ICG [2]. Finally, the intense adsorption peak at 1091 cm^{-1} is due to the CO stretching of PVA, while the nearby features at 1066 and 1027 cm^{-1} belong to the CO stretching of chitosan [3].

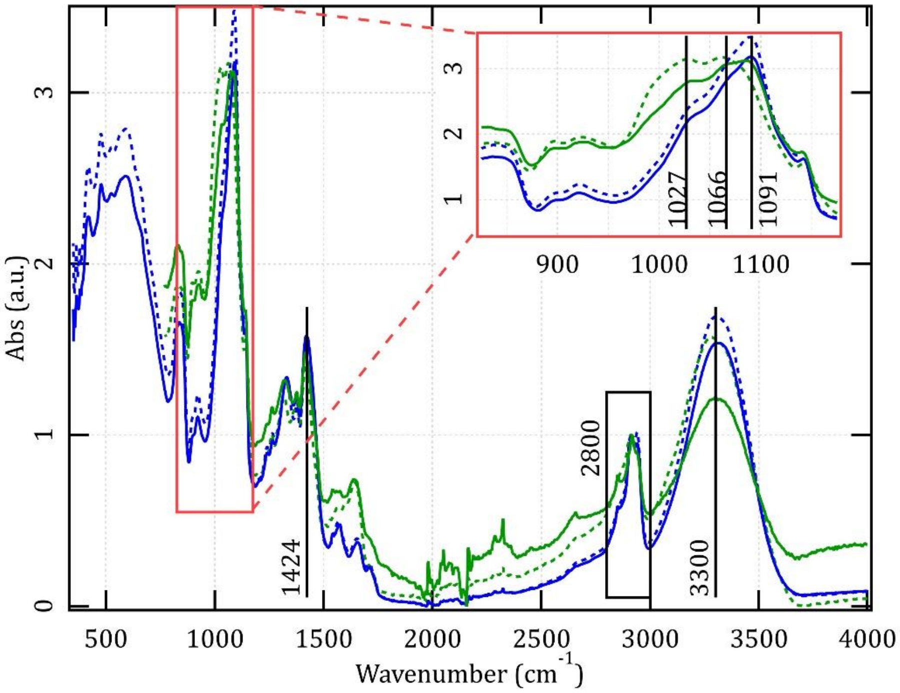

Figure S1. FTIR spectra of as-spun (dotted) and cross-linked (continuous) samples before (blue) and after (green) hydration.

In order to compare the intensity of some bands of interest, all spectra were normalized by the adsorption peak at 2910 cm^{-1}, which is the CH vibration of the backbone. With respect to the as-spun sample before hydration (blue dotted line), the cross-linked one before hydration (blue continuous line) exhibits a clear decrease in the bands assigned to the NH and OH vibrations at 3300 cm^{-1}, due to their involvement in the crosslinking reaction with glutaraldehyde (GLA). The same behavior is visible also in the as-spun sample after hydration (green dotted line) and the cross-linked one after hydration (green continuous line). Moreover, the comparison between the as-spun sample before (blue dotted line) and after hydration (greed dotted line) reveals a significant reduction of the adsorption peak at 1091 cm^{-1} associated to PVA, and a clear prevalence of chitosan with its distinctive double peak at 1066 and 1027 cm^{-1}. This indicates that soaking in water induces a partial dissolution of PVA. Conversely, in the cross-linked sample after hydration (green continuous line), the persistence of the feature at 1091 cm^{-1} implies that the crosslinking process is useful to stabilize the mat by inhibiting the dissolution of PVA.

X-ray diffraction of the cross-linked electrospun films

Wide-angle Powder X-Ray Diffraction (PXRD) patterns of the cross-linked films, pure chitosan powder and PVA pellets were acquired at room temperature with an XPERT-PRO diffractometer from Panalytical (Almelo, the Netherlands) using K_α-radiation ($\lambda = 1.54187$ Å) and a parabolic focusing mirror for Cu-radiation. The spectra of all compounds were collected with a zero-background Si wafer as sample holder in the 2θ interval from 4 to 60° with step size of 0.0263°. The crystallite size of the samples was determined by the Debye-Scherrer method [4], using LaB_6 as reference material.

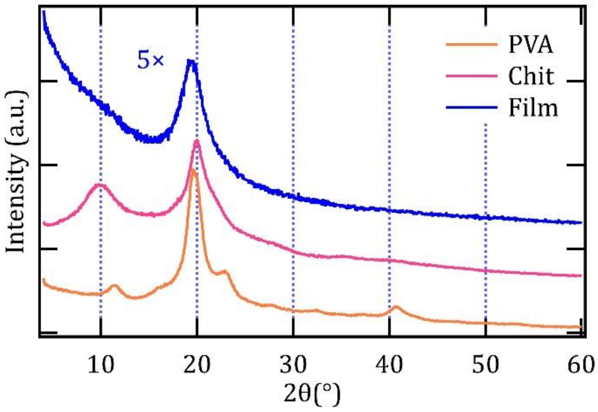

Figure S2. PXRD spectra acquired at room temperature for a cross-linked film, chitosan and PVA.

The PXRD pattern acquired for the cross-linked film displays one broad Bragg reflex centered at 19.43° (2θ). A comparison of the latter spectrum with those acquired from pure chitosan powder and PVA pellets confirms the homogeneous composition of the composite film, which is not a simple mechanical mixture of separate microphases of its principal components. The crystallite size of the cross-linked film of 30 Å is significantly smaller than those of chitosan (35 Å) and PVA (53 Å).

Cytotoxicity of the electrospun films upon cross-linking

The cytotoxicity of the cross-linked films was assessed on adult human dermal fibroblasts (HDFs) as a relevant model in the context of skin lesions. Cells were counted using a Neubauer chamber (Karl Hecht Assistent, Sondheim vor der Rhön, Germany). 80 × 10^3 cells were seeded in 24-well plates (Greiner Bio-One Italia, Milan, Italy) and maintained under standard conditions (37°C, 5% CO_2). Specimens of films treated with vapors of GLA were cut with a 6-mm surgical puncher and co-incubated with the HDFs for 72 hrs. Cellular viability was quantified using Cell Counting Kit-8 (CCK-8) assay (Sigma-Aldrich, Milan, Italy), which implements WST-8 as a colorless reagent that becomes orange when bio-reduced to formazan by mitochondrial dehydrogenases. The CCK-8 assay was used according to the instructions provided by the manufacturer. The optical absorbance at 450 nm (reference at 630 nm) was read using an automatic microplate reader (Multiskan FC Microplate Photometer, Thermo Fisher Scientific, Milan, Italy). Data were processed with SkanIt software.

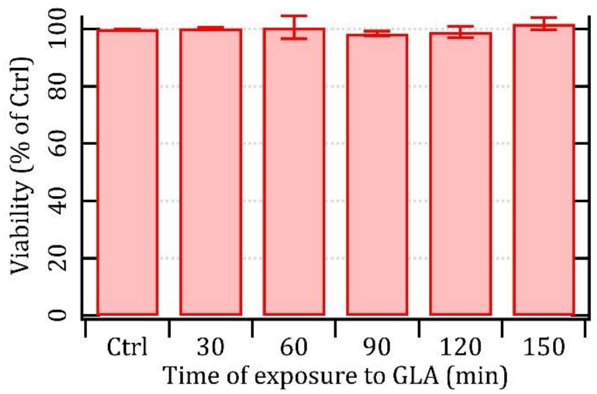

Figure S3. Cellular viability measured after 72 hrs in the presence of 28-mm^2 specimens of electrospun films treated with GLA for different intervals from 30 to 150 min. The control was cells left without any film. All measurements were done in triplicate.

We found no sign of cytotoxicity even in the case of films treated with GLA for 150 min.

References:

[1] Rosli, N.; Yahya, W.Z.N.; Wirzal, M.D.H. Crosslinked chitosan/poly(vinyl alcohol) nanofibers functionalized by ionic liquid for heavy metal ions removal. *Int. J. Biol. Macromol.* **2022**, *195*, 132-141.

[2] Jung, B.; Anvari, B. Synthesis and characterization of bovine serum albumin-coated nanocapsules loaded with indocyanine green as potential multifunctional nanoconstructs. *Biotechnol. Prog.* **2012**, *28*, 533-539.

[3] Fernandes Queiroz, M.; Melo K.R.; Sabry, D.A.; Sassaki, G.L.; Rocha, H.A. Does the use of chitosan contribute to oxalate kidney stone formation? Mar. Drugs **2014**, *13*, 141-158.

[4] Holzwarth, U.; Gibson, N. The Scherrer equation versus the 'Debye–Scherrer equation'. *Nat. Nanotechnol.* **2011**, *6*, 534.
